# Experiences of using a participatory action research approach to strengthen district local capacity in Eastern Uganda

**DOI:** 10.1080/16549716.2017.1346038

**Published:** 2017-08-31

**Authors:** Moses Tetui, Anna-Britt Coe, Anna-Karin Hurtig, Elizabeth Ekirapa-Kiracho, Suzanne N. Kiwanuka

**Affiliations:** ^a^ Department of Health Policy, Planning and Management, Makerere University College of Health Sciences, School of Public Health (MakCHS-SPH), Kampala, Uganda; ^b^ Sociology Department, Umeå University 901 87 Umeå, Sweden; ^c^ Epidemiology and Global Health Unit, Department of Public Health and Clinical Medicine, Umeå University, Umeå, Sweden

**Keywords:** MANIFEST - Maternal and Neonatal Implementation for Equitable Systems Study, Participatory action research, stakeholders experiences, local capacity, districts, implementation science

## Abstract

**Background**: To achieve a sustained improvement in health outcomes, the way health interventions are designed and implemented is critical. A participatory action research approach is applauded for building local capacity such as health management. Thereby increasing the chances of sustaining health interventions.

**Objective**: This study explored stakeholder experiences of using PAR to implement an intervention meant to strengthen the local district capacity.

**Methods**: This was a qualitative study featuring 18 informant interviews and a focus group discussion. Respondents included politicians, administrators, health managers and external researchers in three rural districts of eastern Uganda where PAR was used. Qualitative content analysis was used to explore stakeholders’ experiences.

**Results**: ‘Being awakened’ emerged as an overarching category capturing stakeholder experiences of using PAR. This was described in four interrelated and sequential categories, which included: stakeholder involvement, being invigorated, the risk of wide stakeholder engagement and balancing the risk of wide stakeholder engagement. In terms of involvement, the stakeholders felt engaged, a sense of ownership, felt valued and responsible during the implementation of the project. Being invigorated meant being awakened, inspired and supported. On the other hand, risks such as conflict, stress and uncertainty were reported, and finally these risks were balanced through tolerance, risk-awareness and collaboration.

**Conclusions**: The PAR approach was desirable because it created opportunities for building local capacity and enhancing continuity of interventions. Stakeholders were awakened by the approach, as it made them more responsive to systems challenges and possible local solutions. Nonetheless, the use of PAR should be considered in full knowledge of the undesirable and complex experiences, such as uncertainty, conflict and stress. This will enable adequate preparation and management of stakeholder expectations to maximize the benefits of the approach.

## Background

Participatory action research (PAR) can be defined as a study design that treats the communities of inquiry as part of the generators of knowledge.[1,2] Groups that utilize PAR attempt to redistribute power relations by working as a team to decide what is researched, how it is researched and its benefits across all stakeholders involved. In such collaborations, communities in which research is being undertaken take a central role in the decision-making, since the PAR approach involves taking local actions to resolve social injustices.[3] Through iterative processes, participants seek to collaboratively identify social problems, adopt potential solutions and devise strategies to overcome challenges. Such processes are promoted within highly respectful and yet deliberately analytical discussions in order to collectively generate lasting solutions to problems.[1–3] By strengthening local capacity and empowering locals, PAR has been lauded as an approach that promotes sustainability of health interventions.[4]

Public health scholars report that weak and unresponsive health systems in low-income countries are an obstacle to sustaining positive health outcomes.[5,6] The PAR approach, as noted above, offers opportunities of making health systems more responsive by building local capacity.[7] Participation of all relevant stakeholders in making a contribution towards improving health outcomes is increasingly advocated for, especially among vulnerable populations.[7,8] Vulnerable communities often lack control over their own health and depend on outsiders to resolve their challenges. PAR engages the vulnerable by empowering them to actively question their situations and develop local solutions to overcome them.[9]

Uganda’s health system faces numerous challenges, including a high burden of infectious diseases, an increasing burden of non-communicable diseases and a persistently high burden of maternal mortality.[10] Because the health system has inadequate financing, management, infrastructure and resources, external agencies such as universities, non-governmental organizations and other donor or development agencies often implement interventions to fill existing gaps.[11] Makerere University College of Health Sciences – School of Public Health (MakCHS-SPH) is one such external agency within the country. To strengthen the health system at district level, a research team (at MakCHS-SPH), in collaboration with three local districts in Eastern Uganda, used the PAR approach to design and implement a maternal and neonatal health project. The project was called Maternal and Neonatal Implementation for Equitable Systems (MANIFEST). The MANIFEST project aimed to improve and sustain maternal and neonatal health outcomes in part by strengthening the capacity of the local district health system, thereby making it more responsive.

The PAR approach has been applied widely within the field of health, usually to deal with social problems and challenges.[12] Public health scholars report the use of PAR as mainly desirable and positive.[13,14] Stakeholders report the approach as empowering, engaging, building an atmosphere of trust among them and promoting collective responsibility.[4,15,16] Nonetheless, cases of conflict among stakeholders, mistrust, ambiguity and a lack of scientific rigor, are some the shortcomings associated with PAR.[17,18]

Documentation of the PAR approach mainly occurs in high-income countries, where it has been used to address social problems such as mental health, educational justice and community development.[3,19,20] Similar documentation in low-income countries (LICs) is much less, especially in sub-Saharan Africa, although it is worth noting that the use of PAR in LICs is widespread in community-based interventions.[2,3] This study bridges this gap by increasing documentation of the use of PAR in LICs. The study focused on looking at experiences of stakeholders at higher levels of authority within district level health systems, rather than those of community level stakeholders, which have been studied more extensively.[21,22] Thus, the aim of this article is to explore stakeholders’ experiences of the PAR approaches about strengthening local health systems capacity, such as management, which is essential for sustaining effective interventions to improve maternal and neonatal health outcomes.

## Study context

**Figure 1.**
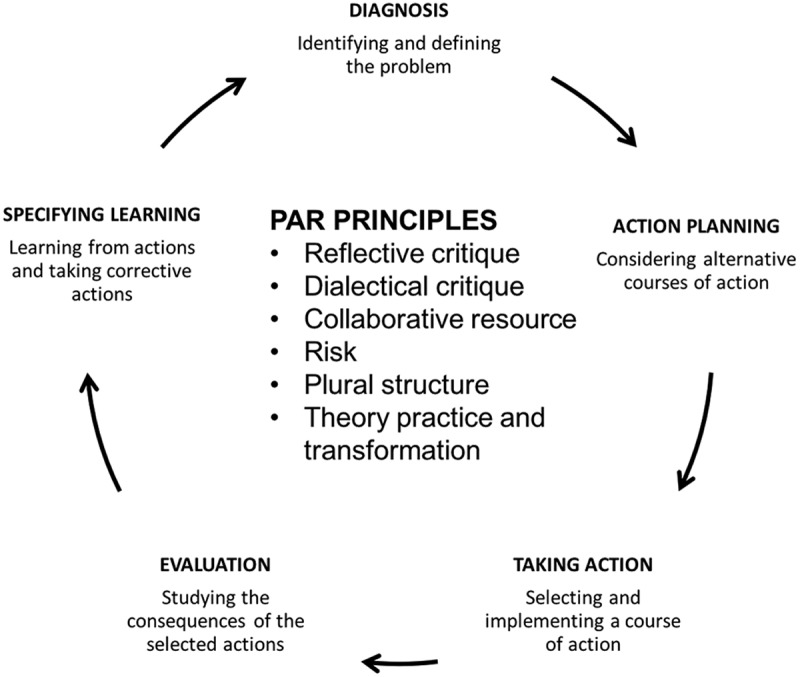
Gerald Susman’s PAR model. The PAR approach is a cyclic collaboration of stakeholders to resolve a given social problem within specific principles, as shown in the center of the figure.

This study was embedded in the MANIFEST study whose detailed design is provided elsewhere.[23] MANIFEST was implemented following Gerald Susman’s PAR cycle.[1] While this was a pragmatic choice, the different frameworks agree on PAR being an iterative process based on principles that promote local capacity building geared towards a collective social change.[1,2,24] According to Susman, the PAR cycle has five phases (Figure 1): problem diagnosis, action planning, taking action, evaluation and specifying learning achieved. The cycle repeats itself with a refinement of the problem or a new one.[25] In addition, the local communities or stakeholders usually take the lead position in the entire process to build local capacity.

At the center of the PAR cycle are principles that build and strengthen communities and systems through the inclusive nature of dialog and actions made at various levels.[14]

Reflexive critique is about providing stakeholders with the opportunity to reflect upon whatever problems and judgments they make about a social problem. This reflection happens within respectful and critical dialog, which is the principle of dialectical critique. Collaborative resource is about different participants making a valued contribution to the process of research and social change. Risk, on the other hand, speaks of the conflict that the approach could create while seeking to challenge or change the status quo. Nonetheless, such risk is essential for change to happen. The plural structure principle states that there are multiple views and options in dealing with social problems. Accordingly, these multiple views should be made explicit to allow informed decision-making. Lastly, theory, practice and transformation attests to the fact that in any setting, people’s actions are based on tacitly-held assumptions that inform their actions upon which theoretical knowledge is built and enhanced.[1]

### The PAR cycle within the MANIFEST study

Phases one and two of the PAR process were conducted in the formative stage (January–December 2012) of the project. Stages three to five were added on to the cycle on a quarterly basis during the project implementation stage (January 2013–December 2015). MANIFEST was implemented in three eastern Uganda districts, namely Kamuli, with a population of 486,319 people; Pallisa with 386,890 and Kibuku with 202,033 people; all of which are under Uganda’s current system of decentralized governance.[26]

### Stakeholders involved in MANIFEST

The stakeholders involved in MANIFEST were grouped into four broad categories. **Community stakeholders** included representatives of adult women and men, council leaders, local transporters and savings groups. **Sub-county level stakeholders** were religious, traditional, political and administrative leaders; select technical staff; health workers and health facility managers. The **district level stakeholders** involved political and administrative leaders, health workers, district health managers, select technical staff, religious leaders and representatives of non-governmental organizations. The **MakCHS-SPH researchers** were a multi-disciplinary team that included medical and public health specialists, social scientists, statisticians, economists and development experts.

### Phase one: diagnosis

Different stakeholders were engaged to identify constraints to achieving favorable maternal health outcomes. Problems identified included low utilization of maternal and neonatal health services, poverty, low male involvement in reproductive health issues, lack of transport to health facilities, geographical inaccessibility of health facilities, rude health workers, inadequate skills of health workers and managers, and frequent stock-outs of essential drugs and supplies in the health facilities.

### Phase two: action planning

The different stakeholders arrived at a set of solutions through an iterative process of consultation and reaching agreement. The MANIFEST research team, which included MakCHS-SPH researchers and select district health managers, then embarked on a process of designing the intervention package that was later implemented in Phase Three. The designed intervention had two components: community mobilization and sensitization and quality improvement of services.

The community mobilization and sensitization component focused on stimulating demand for safe maternal health services by increasing women’s access to savings, transport means and needed knowledge. This was done through home visits and community dialogs undertaken by community health workers (CHWs) and radio sensitizations/mobilization. The quality improvement involved training primary healthcare providers and managers on maternal health services, with emphasis on critical clinical and managerial skills, respectively. Similarly, support supervision and mentorship which were supported by external experts working closely with local terms was undertaken. And a biennial health worker symposium was held to share experiences and to reward outstanding performance. In addition, the use of PAR was meant to build local capacity to strengthen the health system by engaging health managers, other political and technical staff in the running of the project at district level.

### Phase three: taking action

The project was implemented within the existing health systems,[26] with district health teams (DHT) or managers leading its coordination and implementation of activities. Politicians, administrators and opinion leaders delivered radio programs to sensitize communities, harnessed political support, monitored implementation, mobilized local resources and engaged in discussions aimed at being responsive to maternal health issues. The MakCHS-SPH research team provided technical and financial assistance to the district stakeholders.

### Phase four: evaluation

Quarterly review meetings were held at community, sub-county, district and research team levels, culminating in strategies identified and undertaken in phases 1, 2 and 3. In addition, baseline, midline and endline surveys were conducted to evaluate the project. Community dialogs and health worker symposia were also used as a platform to track progress and identify constrains. Results from all these were presented at stakeholder review meetings held at different levels. Here discussions aimed at encouraging positive performance and strengthening weak links were undertaken to inform the decision-making processes.

### Phase five: learning

During the quarterly review meetings, lessons were drawn to better understand obstacles and modify action plans accordingly.

## Methods

### Study design

The informants’ subjective experiences of using PAR were examined through a qualitative study design. A qualitative design provided greater and deeper insights into the understanding of how PAR can be used to implement health systems strengthening interventions.

### Selection of informants

Participants were purposively selected from a pool of stakeholders who were actively involved in the MANIFEST project at district level. These included politicians, administrators, health managers and MakCHS-SPH researchers. The administrators and health managers are civil servants of the districts, while the politicians are democratically elected leaders who have a renewable five-year term of office. And lastly, the MakCHS-SPH researchers were the external implementers of the MANIFEST project.

### Data collection

MT carried out semi-structured individual interviews and a focus group discussion (FGD) using open-ended guides. The data from the informants were collected until a point of saturation at the 16th interview was reached.[27] In addition to facilitating the FGD discussion with MakCHS-SPH researchers, MT made his own personal reflections of the PAR approach, since he was one of the researchers. With permission from the study participants, MT audio-recorded interviews and FGDs to make sure that these were documented in their entirety. Table 1 provides a summary of the data sources.

**Table 1.** Summary of respondents and data collection approaches.

Data source	Data collection methods	
Interviewees	Semi-structured interviews	Focus group discussions
Health managers	11	
Politicians	4	
Administrators	3	
MakCH-SPH researchers		1 (number of participants was 7)
Total	**18**	**1**

### Data analysis

The qualitative content analysis process, led by MT, was both inductive and deductive.[28] Prior to the inductive process, MT listened to the audio recordings before transcription to get a general understanding of the participants’ experiences, and after the transcription, to validate the transcripts and get more familiar with the data. Sections of the transcript relevant to the study of stakeholder experiences were identified.

During the inductive process of analysis, coding was carried out to the identified sections of the transcripts to create meaning.[29,30] The meanings were then categorized through a process of identifying relationships between them. MT shared codes and categories with the other authors through an iterative process, which eventually yielded agreement on the final codes. The process yielded four categories that captured how stakeholders experienced the PAR approach. The categories included: stakeholder involvement, being invigorated, risk of wide stakeholder involvement and balancing wide stakeholder involvement. ‘Awakened’ was found to be the overarching category of the experiences. The different stakeholders as described in the results section experienced all of the four categories uniquely. Table 2 provides an example of the movement from text to categories during the inductive data analysis process.

**Table 2.** Example of movement from meaning units to categories during the analysis process.

Text (meaning unit)	Selected open codes	Sub categories	Category
In the study design, I took part in the baseline data collection. We also participated in the dialogs in the communities, understanding their problems and then trying to come up with possible solutions together. Beyond that, after we were successful in acquiring the grant, I have been involved in the capacity building of the communities both at the community level and the capacity building of health workers. I know the communities now, the politicians and everyone one – we know each other. You find that everyone is involved and we are happy. I can tell you people really own this project. We all know what is going on and we want to make sure the project succeeds.	Making a contribution; Getting involved; Being consulted; Considered; Being valued; Being inclusive; Being heard; Involving others; Dialoging; Connecting with others; Owning; Pleased; Knowing; Taking responsibility	Engaged; Valued; Responsible ownership	Stakeholder involvement

In the deductive analysis process, the categories were reflected upon to place the unique experiences of different stakeholders into the five stages of the Susman’s cycle of PAR. These included: the diagnosis stage, action planning, taking action, evaluation and learning as described in the background of this paper and reflected upon in the description of the results in the results section. Finally, eight informants and five of the seven FGD participants reviewed the results as a means of validation.

## Results

**Figure 2.**
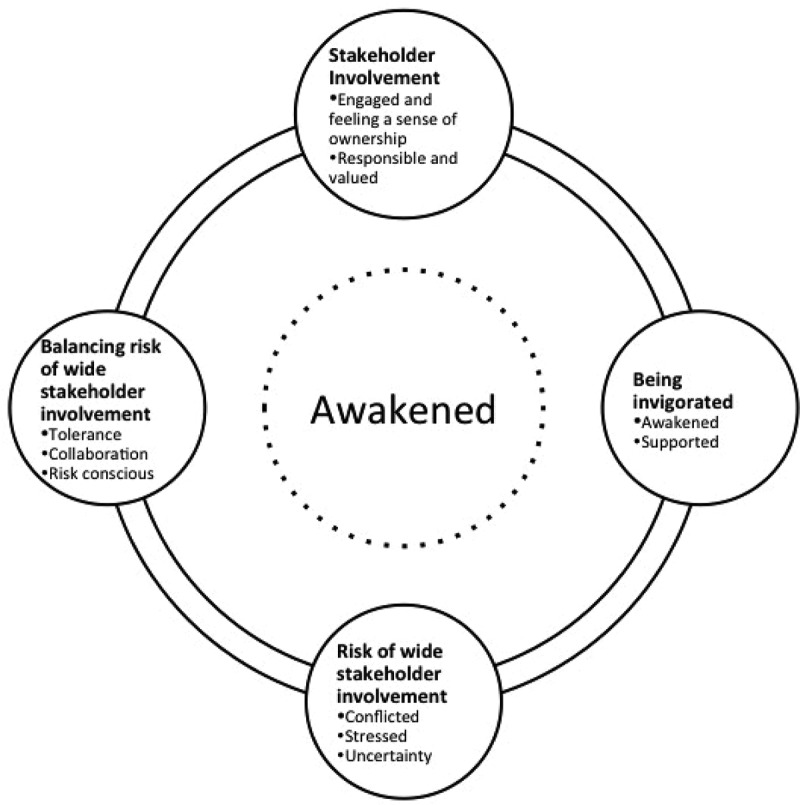
Visual representation of the stakeholder experiences. The stakeholder experiences were found to revolve around being awakened and happened in a sequential manner. Starting with involvement, stakeholders felt invigorated next; this however came with risks of multi-stakeholder involvement, which prompted action to balance the risks.

Four interrelated and sequential categories describe the stakeholders’ experiences of using PAR. ‘Awakened’ was found to be the overarching category around which the four categories of the stakeholder experiences revolved. The approach was reported to have stirred up local stakeholders to create change in a manner that was previously not experienced with other non-participatory approaches. In terms of sequence, stakeholder involvement was experienced as essential for being invigorated. In addition, with the wide stakeholder involvement came risks of engagement that had to be managed through the category of balancing risks of wide stakeholder engagement. Figure 2 is a visual representation of the stakeholder experiences of using a PAR approach.

### Stakeholder involvement

This category referred to how stakeholders experienced their involvement in the MANIFEST intervention. Overall, all stakeholders felt engaged, had a sense of ownership, responsibility and felt valued. These experiences varied at different stages of the intervention.

#### Engaged and feeling a sense of ownership

In terms of engagement, the health managers, politicians and administrators noted that they were consulted and their views considered during all five stages of the PAR cycle. According to the MANIFEST design, they collectively identified problems, solutions, acted and reviewed progress and learnt as a team. About the engagement, two informants remarked:

'I was involved in designing the program, I took part in consulting the communities and I am still involved in the project activities. So, I have been engaged before and after we got the grant.' (A health manager)

'I am involved in MANIFEST, for example, I take part in the radio talk shows and MANIFEST provides us with some airtime, where we sensitize our mothers and men. We talked about how they supposed to treat pregnant mothers and on how my health workers are can attract these mothers to the facilities.' (A politician)

This level of engagement was viewed as creating a sense of local ownership among all the stakeholders across the five stages of the implementation cycle. A political leader made the remark below as a benefit of engagement.

'For this case where the program brings both political and technical people together, we reach a common understanding and we own the project wholly. So that is very, very good and I want to commend you for this approach, it is very good.' (A politician)

#### Responsible and valued

In terms of responsibility, politicians, administrators, health managers and the MakCHS-SPH researchers all experienced PAR as challenging them to be more reflective, responsible and accountable. This was experienced mainly at the stages of taking action and evaluation, in which the onus to find local solutions, monitor resource use and mobilize local resources was upon the local stakeholders. The quotes below exemplify the responsibility of stakeholders.

'Now the district chairperson goes around mobilizing for health and at funerals he goes saying ‘men get involved’ and it really puts out the message with not only another voice, but a better one. He is now more responsive to the health needs of the community.' (A health manager)

'During MANIFEST, people gave their own views of what they think is good for them and we worked with them, this made them more responsible and accountable.' (MakCHS-SPH researcher 1)

'You are building a leader. If you had come and done everything, then you would leave me the same way you got me. But now you have given me an opportunity to have a hands-on; even if you go away, I will still have these skills that you have been able to impact in me, which will help me continue.' (A health manager)

Given the responsibilities undertaken by the stakeholders, they often felt valued, which meant being accepted and appreciated. All the stakeholders felt appreciated and accepted mainly during the taking action and evaluation phases. These were the phases in which stakeholders actively took part in implementation of activities and reviewed progress, which created opportunities for appreciating one another. In addition, it opened spaces for sharing local knowledge and expertise, while identifying what worked well and or what did not at these stages, respectively.

'For sure, when I am told to go for the radio talks, I feel respected and valued as a leader in the district. I think this is good, thank you.' (A politician)

### Being invigorated

This category referred to awakening of previously dormant structures and stakeholders by being actively involved in the MANIFEST project. Through involving a range of stakeholders, the PAR approach was reported as invigorating. It provided opportunities for all stakeholders to actively make a contribution towards the successful implementation of the project. This was challenging and enriching at the same time, as the stakeholders worked together.

#### Awakened

Politicians, administrators and health managers felt awakened and inspired. They reported that their hidden capabilities and structural potential was rediscovered. This was felt mainly at the stages of taking action and evaluation. According to them, prior to the project, civil servants and other stakeholders at district level were generally used to doing less work, compared to the engagements that the project required. They were also limited in their use of existing resources, as they often thought of resolving challenges through seeking external support. Through these wide stakeholder interactions and involvement in the MANIFEST project, they all felt inspired to perform better, especially during the stages of taking action and evaluation. The reflections below highlight awakening.

'We were like we had gone into slumber. Waking up people, especially adults can be difficult, but this approach woke us up.' (An administrator)

'Yea, I didn’t know that these CDOs (community development officers) also had anything to offer to the health department, but I think you have seen, they are very important with these things of savings for health. MANIFEST has brought many different people together, I think that is good.' (A politician)

#### Supported

In addition to being awakened, the project provided opportunities for stakeholders to support each other. With regular and close involvement and interactions, managers, politicians and administrators reported being supported to implement project activities through a process of learning by doing. They reported this as empowering a feeling that made them more confident about their abilities to deliver specific services. The researchers, on the other hand, reported having their skills of mentoring enhanced. This was attributed to the direct involvement of local stakeholders in the design and implementation of the project as a key principle of PAR. Such involvement was noted to have provided the opportunity to learn how to mentor locals to undertake project activities, rather than the project team spearheading the implementation. Being supported was mainly reported to have occurred during the phases of taking action, evaluation and learning. The following reflections were made regarding feeling supported.

'Working with our colleagues from Makerere really helped me. For example, last time, I had a challenge with using the computer, but you helped me. And these days computers are very important. ' (A health manager)

'Supporting others and stepping aside and letting them do things (and) encouraging them to do things the right way, is an approach that we were not used to, so I think we got better at that. ' (MakCHS-SPH researcher 4)

### Risk of wide stakeholder involvement

The category referred to the limitations of wide stakeholder engagement. The stakeholder at times experienced the approach as creating conflict, stress and a sense of uncertainty.

#### Conflicted

The politicians and administrators sometimes conflicted with the health managers, especially during the taking action and evaluation phases. Some health managers considered politicians and administrators too demanding and found harmonizing of their interests difficult, which was frustrating to the health managers. The politicians and administrators, on the other hand, felt sidelined from project activities and disrespected by the managers, as reflected in the quotes below.

'At the beginning, we were invited, they brought us on board, but when it comes to these small meetings of savings in the communities, we are not invited to open or close them and yet we have the key to open and to close. That is the only challenge I see. So, we should be involved seriously.' (A politician)

'I can tell you, things are different here; these people (health managers) are doing their own thing; even the RDC (Resident District Commissioner) is not happy. For us politicians and administrators, you should come and talk to us, then we see how to be involved, but we just hear that things are happening.' (An administrator)

#### Stressed

The health managers felt that the PAR approach was stressful. They felt torn apart, overburdened and frustrated by the deep involvement in the project implementation, especially at the phases of taking action and evaluation. They had the responsibility to coordinate the implementation and evaluation of project activities at district level, which at times conflicted with their other routine duties and the demands from other projects, hence creating stress. The quote below demonstrates the stressful experience reported by the health managers.

'Actually, you find that you have to report on so many issues. In MANIFEST alone we have many reports, but we also have these other routine reports and some of us work with other partners, which can be very difficult to balance, but we are managing.' (A health manager)

#### Uncertainty

The researchers and health managers felt that the PAR approach created ambiguity of boundaries between stakeholders. This created a sense of powerlessness for both the health managers and researchers, especially during the phases of taking action and evaluation. Powerlessness was felt, especially as managers and researchers tried to ensure the implementation of activities or action points agreed upon during review meetings. Working within the existing system and structures seemed bureaucratic and slowed down the implementation of some activities, since they had little or no control over such processes. The quotes below attempt to reflect the ambiguity and powerlessness reported, respectively.

'My observation is that when you go participatory, you don’t know what the boundaries are. Sometimes you get sucked into the already existing local conflict and somehow you are expected to be mediating and negotiating things, yet you have very little knowledge of their genesis. For instance, when one party reports their District Health Officer (DHO) to you, conflict arises, because you need the good will of the DHO as well.' (MakCHS-SPH researcher 1)

'The challenge of the local structures is that, as researchers, we had very little control over their policies. For example, some sub-counties were saying the groups must pay to be registered, while others were saying no, and their rates also varied. Then at the district level, they also wanted the same groups to register with them at higher rates. As researchers, we simply wanted to get people to start saving and to remove all obstacles. So, somehow, I think the implementation of the savings component was affected. Actually, I saw a conflict of interest.' (MakCHS-SPH research 5)

On the other hand, the health managers thought that the close relationship with the researchers created a sense of insecurity, especially during the initial months of project implementation. The quotation below is taken from the researchers and managers to demonstrate the ambiguity and insecurity felt, respectively.

'People in the district are used to this laissez-faire type of management. We are not used to being supervised, or implementing together with the funders. We only want to get the report to you, so sometimes it felt like I was being watched and that was constraining.' (A health manager)

### Balancing wide stakeholder involvement

Given the risks involved with the wide stakeholder engagement, this category entailed the experiences of the stakeholders in trying to minimize these risks. The stakeholders reported tolerance, teamwork and being risk conscious.

#### Tolerance

Tolerance entailed being patient, courteous and flexible with each other. MakCH-SPH researchers reported exercising patience with local stakeholders. This tended to emerge from the health managers’ limited skills in coordinating and implementing project activities. The researchers exercised patience amidst a need to implement activities as planned, while trying to build local capacity at the same time. The health managers, on the other hand, reported being courteous with the researchers and politicians, given the high demands that the project put on them. Tolerance was especially required during the phases of taking action and evaluation.

'I have really learnt to be patient, you have to be understanding, when you work with district people. You don’t know what to expect from them. I was surprised by the way people work at the district. ' (MakCHS-SPH researcher 2)

'Working with these politicians – you just have to be understanding. Sometimes they are very annoying. I think they just want money, nothing else. You find them complaining all the time that we have not included them in the activities, but honestly, some of the activities don’t require them.' (A health manager)

Flexibility was reported as a useful coping strategy when dealing with different stakeholders, especially given the varied stakeholder interests and expectations. In addition, PAR promoted the reviewing of strategies and making of changes if deemed necessary by the stakeholders, in the spirit of being pragmatic about strategies. Nonetheless the researchers thought this flexibility could have easily led to numerous changes without necessarily exploiting each option fully, as noted here.

'If you are told, that you have the option to change your strategy, you somehow become impatient with things. When they don’t work or when you are faced with a challenge, you quickly take the opportunity to change it. That’s one shortcoming I see with this approach, because we changed many things, now I don’t even know why we made some changes.' (MakCHS-SPH researcher 4)

#### Collaboration

The stakeholders reported that they collaborated with each other as a means of dealing with the risks that arose out of the involvement of varied stakeholders in MANIFEST. By this they meant teamwork, interdependence and building trust among themselves. All the stakeholders felt dependent on each other and worked together to ensure the successful implementation of MANIFEST. Teamwork and interdependency were felt mainly at the phases of taking action and evaluation. Trust, on the other hand, was felt while taking action. The stakeholders agreed that using a PAR approach required teamwork, interdependency and trust among themselves, given the need for different kinds of resources, expertise and knowledge. The quote below illustrates collaboration.

'You see, I had never worked so closely with these politicians, but now we understand and trust each other. They have a part to play with the mobilization and sensitization of the communities and we also have our part.' (A health manager)

#### Risk conscious

For the researchers, implementing the project efficiently and safeguarding themselves against fraud and mismanagement of the project was important. This was informed by the need to be accountable to the funding agency, as well as to complete the project within specified timelines. The researchers were mainly risk-conscious during the phases of taking action and evaluations, which were fully delegated to district managers to coordinate and implement. They noted that because PAR promoted wide stakeholder involvement and tolerance, the importance of a memorandum of understanding (MoU) among stakeholders to help manage expectations and clarify roles was useful.

'An MoU is very important; you have to be strict on certain things. You need to stipulate clearly at the beginning of the project what will be done if things went wrong. I think this really helped us when we had those accountability issues in district X.' (MakCHS-SPH researcher 6)

## Discussion

Four interrelated categories that depicted the sequential stakeholder experiences of the PAR approach were identified in this study. Stakeholder involvement promoted engagement, local ownership and responsibility. With the interactions of different stakeholders, being invigorated (awakened and supported) was experienced. The involvement and interactions brought with them risks and that had to be balanced or managed. In the discussion section, we compare our findings to the Susman’s principles of the PAR approach [1] and their relevance to local capacity building, which is essential for the continuity of health interventions.

Susman thought of the PAR process as a repetitive cycle of problem and solution identification, taking action, evaluating and specifying learning. The stakeholders reported different experiences at each of the Susman’s phases of PAR. The phases of action taking, evaluation and learning were found to be the most involving, relative to diagnosis and action planning. While this is expected, a critical review of skill sets needed during these phases is essential to fully harness the benefits of the approach, as well as ensure robustness of the phases of diagnosis and action planning. This cycle, according to Susman, is hinged on principles of reflectiveness and respectful dialog, collaboration, risk taking, use of multiple structures and testing of knowledge leading to desired changes.[1]

Under stakeholder involvement, engagement and ownership that were felt throughout the project cycle reflected the positive interactions that involved critical dialog, collaboration and multiple level interactions. Involvement of various stakeholders at different levels of the society yielded local ideas, experiences, and resources that facilitated the implementation of the MANIFEST intervention. Engagement is a known way of promoting local ownership, which facilitates local capacity building and continuity of interventions respectively.[31,32] With such a high level of involvement, a sense of responsibility was experienced, which was mainly facilitated by the reflective critique principle that was practiced at the stages of taking action and evaluation. Here stakeholders critically considered their courses of action, and apportioned each other specific responsibilities to ensure project success.

Being invigorated was a consequence of the wide stakeholder interactions, which similarly mirrored the framework’s principles of concerted resource, risk and thoughtful dialog. These principles were noted to have enabled the awakening and re-functionalizing of previously dormant stakeholders and structures of implementation. The strengthening of local capacity and structures has been reported as one of the key benefits of PAR.[32] This was achieved through support mechanisms that were built within the interactions of the different stakeholders, which encouraged learning by doing and building of confidence within local stakeholders and structures. The principle of testing out ideas and transformation was exercised during the quarterly phases of taking action and reviewing progress, respectively. These played an important role in strengthening the local stakeholders’ capacity and local structures of implementation.

By placing the local stakeholders in the lead of the implementation process, the intervention required a high level of involvement and commitment, especially from the health managers. This created learning opportunities for the health managers, but also created situations of conflict with their routine duties and relationships. The stakeholders risked their social relationships and structures by actively being involved in constructive although challenging dialog with each other concerning the subject of improving maternal health outcomes within the local health system. Proponents of these principles note benefits of capacity building and positive change arising from conflict. However, the critics view it as a risk that could strain relationships and therefore undermine implementation of project. [22,33,34] This study indeed registered some discontentment among stakeholders with the high and wide level of involvement.

Related to the above, the risks of wide stakeholder involvement included conflict, stress and uncertainty, experienced especially at the phases of taking action and evaluation. Conflict and stress, which was experienced by health managers, politicians and administrators, attested to the PAR principle of risk. This is critical for challenging the status quo and facilitating change.[1] The constant critical dialoging, reflection and interaction across stakeholders provided an enabling environment/conditions for challenging the status quo. This was facilitated by the fact that the health managers led the implementation of project activities at district level. However, studies undertaken in the health system have often reported such risks as obstacles.[35] This study harnessed the benefits of risk, such as enabling stakeholders to appreciate each other’s interests, strengths and weakness.

Similarly, while the researchers were concerned with the ambiguity of boundaries among stakeholders, the health managers felt too closely monitored by the researchers. Dealing with health systems challenges has often been regarded as complex and uncertain; the PAR approach provided an opportunity to experience and adapt to these challenges.[34,36] To circumvent some of these challenges, the stakeholders reported three ways of balancing the risks of involvement. These were tolerance, collaboration and being risk conscious.

Tolerance has been reported as a major attribute of PAR in other studies.[9] This promotes the cultivation of positive working relationships between differing stakeholders whose collaboration is inevitable for the successful implementation of projects. Collaboration and tolerance are fertile grounds for building local capacity and specific skills facilitated by the close interactions, as noted earlier.[37] The MakCHS-SPH researchers, on the other hand, advocated for MoUs when using participatory approaches. These were viewed as safety nets against abuse that may result from the tolerance, collaboration and flexibility of the approach.

## Methodological considerations

To achieve credibility, only stakeholders who were actively involved in the implementation of this study were selected. Similarly, experiences from the local district level implementers and the external researchers were captured to get variations in perspectives. However, community members were not interviewed in this study because the study focused on exploring experiences of stakeholders at higher levels of authority. Nonetheless, community stakeholders that took part in the MANIFEST study were interviewed and their views documented elsewhere.[38,39]

In addition, a detailed description of the research setting and processes was provided, to enable transferability of the study findings or at least the methods of inquiry. Dependability was ensured through the open approach to the inquiry process and the review of the analysis process by all the authors of this paper. Finally, conformability was attained through sharing the preliminary findings with the stakeholders for review as a validation process.[28]

## Conclusions

The conclusions drawn from this study relate to two main issues: the desirability of the PAR approach and the complexity of stakeholder experiences using it to strengthen health systems. The PAR approach was found to be desirable among stakeholders and created opportunities for enhancing local capacity and increasing chances for sustainability by awakening local potential within stakeholders and structures. For example, stakeholder involvement and interaction stimulated stakeholder responsibility and could be harnessed for learning of specific skills, such as tolerance and collaboration. Additionally, skills for managing present-day health system complexity could be boosted through the interactions and feedback loop promoted by the approach. Specifically, PAR could strategically be harnessed to build local health managers’ capacity to respond to an ever-increasing dynamic and complex health service sector. Such local capacity is essential for a sustained improvement in health outcomes. However, there is a need to study the conditions necessary for successfully using a PAR approach to fully harness its potential and the actual systems improvement achieved using PAR.

Lastly, the study enhances Susman’s framework by bringing to light the differing and complex stakeholder experiences of using PAR. This will enable adequate preparation and the management of stakeholder expectations to maximize the benefits of the approach. For example, making deliberate efforts to be inclusive when implementing projects, by considering stakeholder interests and needs, could enhance the positive experiences of PAR, while reducing ambiguity through making formal agreements between stakeholders and syncing projects to local structures could be useful for minimizing undesirable experiences.

## Paper context

Uganda’s health system at district level is generally weaker and is persistently challenged by poor health outcomes. External stakeholders such as MakCHS-SPH and other donor agencies often intervene with projects to respond to specific health events, such as HIV or disease outbreaks. The need to strengthen local district health systems is therefore important. Understanding how stakeholders with higher levels of authority experienced PAR was therefore deemed essential, since it is known for strengthening local capacity.
